# Dyeing Method and Properties of a Novel Blue Azo-Anthraquinone Reactive Dye on Cotton

**DOI:** 10.3390/molecules24071334

**Published:** 2019-04-04

**Authors:** Bin Shan, Wei Xiong, Shufen Zhang

**Affiliations:** 1 School of Environmental & Municipal Engineering, Qingdao University of Technology, Qingdao 266033, China; 2 State Key Laboratory of Fine Chemicals, Dalian University of Technology, Dalian 116012, China; 11407102@mail.dlut.edu.cn

**Keywords:** azo-anthraquinone dyes, reactive dyes, dyeing properties, dyeing method, high fastness

## Abstract

A novel blue azo-anthraquinone reactive dye was evaluated in the dyeing of cotton by using a dip–pad–steam process. Dyeing method and properties were examined in detail and the results showed that the dyeing method consisting of dye concentration of 25 g/L, sodium carbonate of 12 g/L, dipping time of 3 min and steaming time of 30 min was the most effective when a conventional “one-dip–one-nip” process was used. The fixation of the dyes on cotton could reach up to 93.4%, the wash and rub fastness both reached grade 4 above, and the light fastness reached grade 4–5 above. Such colored cotton showed very close colorimetric properties.

## 1. Introduction

Recently, azo-anthraquinone dyes have received much attention because of their attractive advantages, including high tinctorial strength and excellent light stability. They have been developed for ink jet printing, polyvinyl alcohol (PVA) polarizing films, new nonlinear optical materials, and dyeing cotton fabric [1,2,3,4]. A great deal of work has been carried out about the incorporation of anthraquinone structure, such as 1-aminoanthraquinone, 2-aminoanthraquinone, 1-amino-4-bromoanthraquinone-2-sulfonic acid derivatives, 1,5-diamino-anthraquinone, and 2,6-diamino-anthraquinone derivatives into the azo chromophore to get azo-anthraquinone dyes [5,6,7,8,9,10,11,12]. All these dyes possess high light fastness and their colors range from yellow to red and assorted green.

However, the reported researches about the azo-anthraquinone dyes mainly focused on acid and disperse dyes, but rarely on reactive dyes. In contrast, reactive dyes are extensively used in the textile industry since they can be linked onto the fibers through covalent bonds, and the dyed fibers have high fastness to wet treatment [13,14,15,16,17,18]. In addition, compared with 1,5- and 2,6-disubstituted and other monosubstituted anthraquinone derivatives, 1,4-disubstituted anthraquinone derivatives with lower energy gap between HOMO and LUMO of the molecular systems are more prone to get deep color shades [19,20,21].

**Figure 1:**
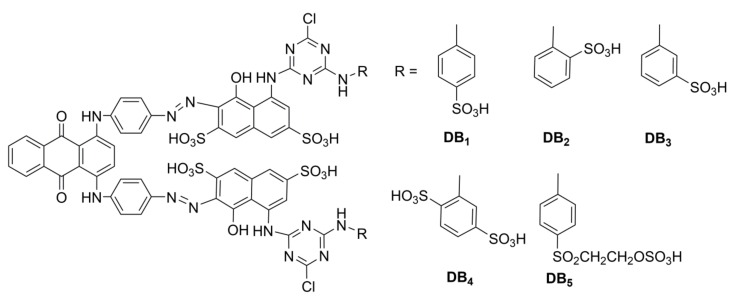
Structures of azo-anthraquinone reactive dyes.

Herein, we report an azo-anthraquinone reactive dye with deep blue color shade synthesized using 1,4-bis((4-aminophenyl)amino) anthrancene-9,10-dione as a diazo component, and 1-amino-8-naphthol-3,6-disulfonicacid (H-acid) derivatives as coupling components. The structures of azo-anthraquinone reactive dyes are shown in Figure 1. The azo-anthraquinone reactive dyes were applied for dyeing cotton fibers by using a dip–pad–steam process. Dyeing method of the dyes on cotton were investigated in detail and dyeing properties comprising wash fastness, rub fastness, and light fastness were examined—as well as colorimetric properties.

## 2. Results and Discussion

### 2.1. Dyeing Method of Azo-Anthraquinone Reactive Dyes on Cotton

**Figure 2:**
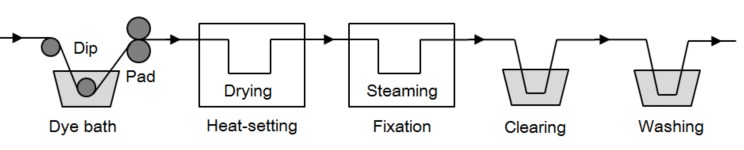
Dyeing process of azo-anthraquinone reactive dyes.

A dip–pad–steam process was used for dyeing of cotton fibers with the azo-anthraquinone reactive dyes, and the dyed fabrics were treated by the clearing and washing process in order to remove unreactive dyes (Figure 2). Since the dye molecule contains 2–4 reactive groups, it can easily react with the cotton fibers under suitable conditions, and its reactivity towards the cotton fibers was especially high, resulting in high dye fixation [22]. Furthermore, the azo-anthraquinone reactive dye shows certain higher solubility in water, owing to the 6–8 sulphonic groups in the molecule. The dye structure shows a planar configuration of the anthraquinone core part in the dye molecule [23], which exhibits lower substantivity to cotton fibers. For this kind of dye to finish cotton, the “one-bath–one-step” dyeing method was proved to be a suitable process [24]. Thus, in this study, dyeing conditions were optimized for achieving high dye fixation. The dyed fiber properties were also tested to see whether they had reached the standard requirements or not. K/S value was assessed as an important parameter of dyeing properties, which could provide a measure of the color depth of the dyed fabric. The optimal conditions were determined by examining the influence of Na_2_CO_3_ concentration, dye concentration, dipping time, number of dip and nip, and steaming time on dye fixation and K/S value of the dyed cotton. The azo-anthraquinone reactive dye **DB_1_** was employed as the model dye for investigation in detail. With all the results, the dyeing method can be evaluated comprehensively.

### 2.2. Effect of Na_2_CO_3_ Concentration on Dyeing Properties

**Figure 3:**
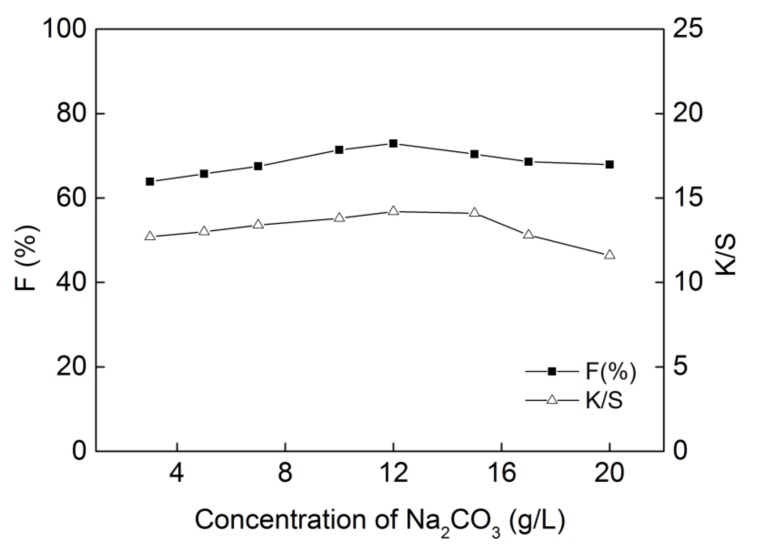
Effect of Na_2_CO_3_ concentration on dyeing properties of **DB_1_** (dye concentration of 40 g/L, dipping time of 3 min and steaming time of 15 min with “one-dip–one-nip” process).

Na_2_CO_3_ was used in the dyeing process to ionize the cellulose hydroxyl groups for reactive dye fixation. In the “one-bath–one-step” dyeing method, Na_2_CO_3_ was added directly to dye bath and the results obtained for dyeing properties with differing amounts of Na_2_CO_3_ are shown in Figure 3. From the results, it can be seen that the fixation and K/S value of the dyed cotton both increased when the concentration of Na_2_CO_3_ was increased from 3 g/L to 12 g/L. When the concentration of Na_2_CO_3_ was further increased to 20 g/L, the fixation on cotton and K/S value both decreased. Na_2_CO_3_ was added to the dye bath based on the fact that the alkaline conditions would promote the nucleophilic substitution of the dye and fiber during the fixing process, while a much higher concentration of Na_2_CO_3_ would cause greater hydrolysis of the dye [25]. Accordingly, 12 g/L of Na_2_CO_3_ was selected to be used in the following investigation.

### 2.3. Effect of Dye Concentration on Dyeing Properties

**Figure 4:**
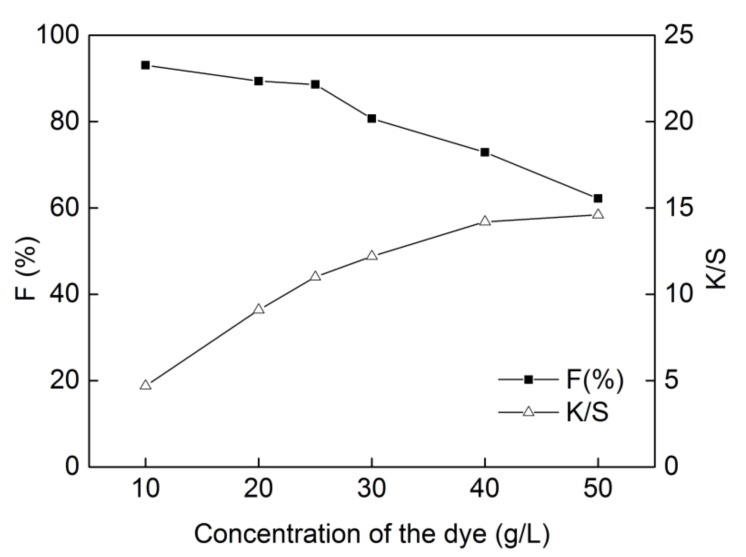
Effect of dye concentration on dyeing properties of **DB_1_** (sodium carbonate of 12 g/L, dipping time of 3 min and steaming time of 15 min with “one-dip–one-nip” process).

Dye concentration influenced both dye fixation and color strength of the dyed cotton in the dyeing process, as shown in Figure 4. As is shown, with the increase of dye concentration from 10 g/L to 50 g/L, K/S value of the dyed fabric increased from 4.7 to 14.6—while the dye fixation declined obviously from 93.1% to 62.2%. Due to the steric effect of the large molecule and planar configuration structure of the dye, many dye particles were adsorbed on the surface of cotton, and the hydroxy sites of the cotton fibers were not enough for dye exhaustion with increasing dye concentration. When dye concentration reaches 25 g/L, K/S value of the dyed fabric can reach a 1:1 color depth according to ISO 105-A01-2010. Therefore, a dye concentration of 25 g/L was selected as relatively suitable for dyeing.

### 2.4. Effect of Numbers of Dip and Nip on Dyeing Properties

**Figure 5:**
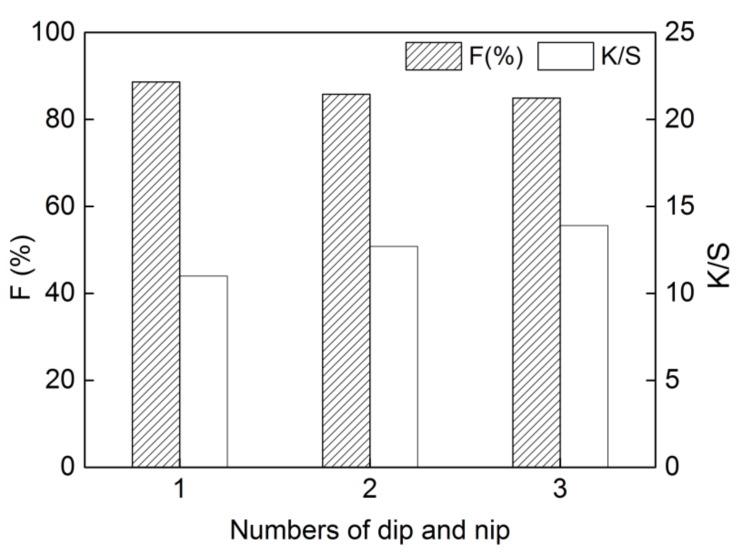
Effect of numbers of dip and pad on dyeing properties of **DB_1_** (dye concentration of 25 g/L, sodium carbonate of 12 g/L, dipping time of 3 min and steaming time of 15 min).

The results of the dyeing process through “one-dip–one-nip”, “two-dip–two-nip”, and “three-dip–three-nip” operations are presented in Figure 5. It shows that the fixation decreased slightly from 88.6% to 84.9%, and K/S value of the dyed cotton increased from 11.0 to 13.9 when the numbers of dip and nip were increased. With the increase in numbers of dip and nip, the color yield could be effectively improved. However, more dyes adsorbed on the surface of cotton could not penetrate the fiber inside, which were not linked to the cotton fibers, so the fixation decreased slightly. Accordingly, a “one-dip–one-nip” padding was selected for dyeing.

### 2.5. Effect of Dipping Time on Dyeing Properties

**Figure 6:**
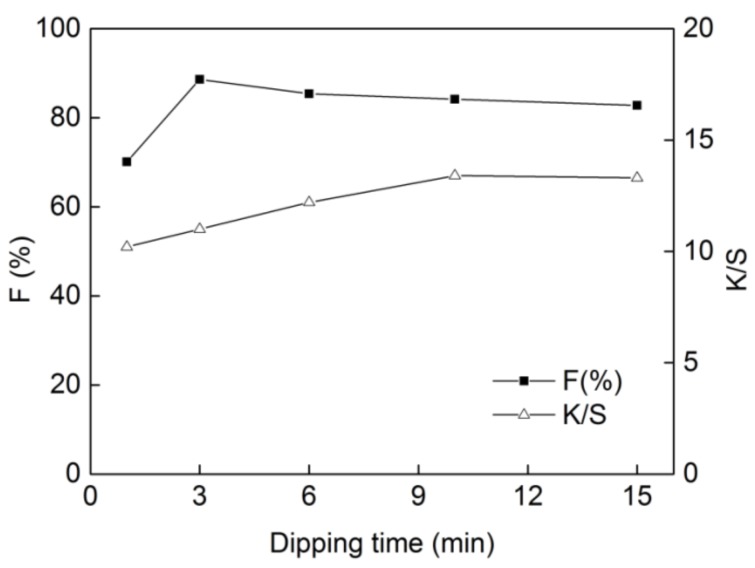
Effect of dipping time on dyeing properties of **DB_1_** (dye concentration of 25 g/L, sodium carbonate of 12 g/L and steaming time of 15 min with “one-dip–one-nip” process).

The effect of the dipping time on dye fixation and K/S value was also investigated, as shown in Figure 6. The results indicate that when the dipping time was increased from 1 min to 3 min, the fixation increased from 70.1% to 88.6% and K/S value increased from 10.2 to 11.0, respectively. If the dipping time was below 3 min, dye molecule cannot penetrate the fiber inside, so lower levels of fixation and K/S value were obtained. However, no improvement in dye fixation was found when the dipping time further increased from 3 min to 15 min, owing to the equilibrium of absorption-desorption of the reactive dyes on cotton. So, the dipping time of 3 min was selected for dyeing.

### 2.6. Effect of Steaming Time on Dyeing Properties

**Figure 7:**
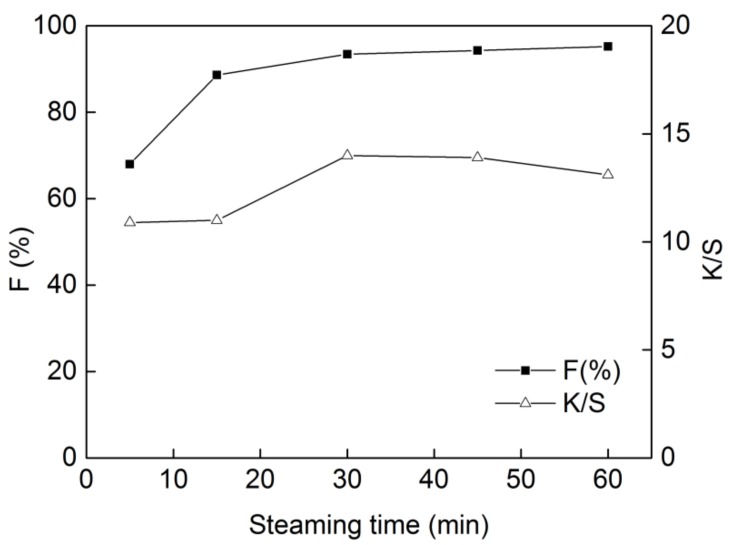
Effect of steaming time on dyeing properties of **DB_1_** (dye concentration of 25 g/L, sodium carbonate of 12 g/L and dipping time of 3 min with “one-dip–one-nip” process).

The steaming time in the fixing process had a great effect on dye fixation and K/S value of dyed cotton, as shown in Figure 7. It was found that when the steaming time reached 30 min, the plot reached a plateau at a fixation of 93.4% and K/S value of 14.0. At steaming time of less than 30 min, a portion of reactive dyes are not linked to the fiber, therefore the fixation can never reach 90%. Whereas, the dye fixation was almost unchanged after 30 min when the reaction of the dyes and fibers was complete, so it was not necessary to prolong the steaming time further. Therefore, the steaming time of 30 min was selected.

### 2.7. Fixation and Fastness Properties

Cotton fibers were dyed and fixed with the azo-anthraquinone reactive dyes using a dip–pad–steam process in the optimum dyeing conditions. Table 1 shows the fixation, colorimetric and fastness properties of the cotton colored with azo-anthraquinone reactive dyes.

The fixation of the azo-anthraquinone reactive dyes **DB_1_**, **DB_2_**, **DB_3_**, **DB_4_,** and **DB_5_** on cotton fibers reached 93.4%, 88.4%, 91.8%, 88.2%, and 89.9%, respectively. The color strength and other color parameters of brightness (L *), redness–greenness (a *) yellowness–blueness (b *), color saturation (C *), and hue angle (h *) were compared [26]. The K/S value of the dyed cotton reached a 1:1 color depth according to ISO 105-A01-2010, and the azo-anthraquinone reactive dyes **DB_1_**, **DB_2_**, **DB_3_**, **DB_4_,** and **DB_5_** had basically the same L *, C *, and h *, demonstrating a very close color shade of dyes with the same chromophore. Moreover, a * and b * values of the azo-anthraquinone reactive dyes close in color did not differ much. The wash fastness and dry and wet rub fastness of the dyed cotton all reached grade 4 above. Furthermore, the light fastness reached grade 5, indicating outstanding performance in terms of color fastness. All these features brought very promising future applications for azo-anthraquinone reactive dyes.

## 3. Materials and Methods 

### 3.1. Materials

1,4-Bis((4-aminophenyl)amino)anthrancene-9,10-dione was prepared according to a previous study [23]. Cyanuric chlorine, 2-aminobenzenesulfonic acid, 3-aminobenzenesulfonic acid, 2-aminobenzene-1,4-disulfonic acid, 2-((4-aminophenyl)sulfonyl)ethyl hydrogen sulfate, and 1-amino-8-naphthol-3,6-disulfonicacid (H-acid) were obtained from Zhejiang Shunlong Chemical Co. (Shaoxing, China). 4-Aminobenzenesulfonic acid was purchased from Tianjin Damao Chemical Reagent Co. (Tianjin, China). Emulsifier OP-10 (alkyl phenol polyoxyethylene ether) was purchased from Sinopharm Chemical Reagent Co., Ltd. (Shanghai, China). Bleached, desized, and mercerized 100% cotton (170 g/m^2^) was purchased from Shandong Qisai Textile Co., Ltd. (Zibo, China). All other chemicals used in this study were of synthetic grade.

### 3.2. Synthesis of the Dyes

The azo-anthraquinone reactive dyes **DB_1_**, **DB_2_**, **DB_3_**, **DB_4_,** and **DB_5_** were synthesized according to our previous study [23].

Firstly, 2.40 g of 1,4-bis((4-aminophenyl)amino)anthrancene-9,10-dione was dissolved in 2.5 mL of hydrochloric acid (37%, *w*/*w*) and 50 mL of water. After it had been cooled to 0–5 °C, 1.45 g of NaNO_2_ was added into the solution. The reaction mixture was stirred for 30 min, and Erich reagent was used to detect the completion of diazotization to give the solution of the diazo salt. Secondly, 1.90 g of cyanuric chloride and 20 g of ice cubes were stirred for 30 min, and then 0.01 mol of 4-aminobenzenesulfonic acid (2-aminobenzenesulfonic acid, 3-aminobenzenesulfonic acid, 2-aminobenzene-1,4-disulfonic acid, or 2-((4-aminophenyl)sulfonyl)ethyl hydrogen sulfate) was added. The reaction mixture was stirred for 1 h at 0–5 °C and pH 4–5 until no chromogenic reaction to the Erich reagent was detectable. After the reaction was completed, 4.0 g of H-acid was added. The reaction mixture was stirred for about 3–4 h at 25–30 °C and pH 5–5.5, and thin layer chromatography (TLC) was used to monitor the completion of the condensation reaction (n-PrOH:i-BuOH:EtOAc:H_2_O, 2:4:1:3, *v*/*v*). Finally, the solution of diazo salt made above was added slowly to the solution of condensation product and the reaction was performed at room temperature and pH 7–8. When the reaction was completed, potassium acetate (15% *w*/*v*) was added and the solid blue product was isolated, then filtered and dried in a vacuum.

UV-Vis, IR, MS, and ^1^H-NMR were used for spectral and structural characterization of these dyes, and the absorption maximum (λ_max_) of **DB_1_**, **DB_2_**, **DB_3_**, **DB_4_,** and **DB_5_** in water was 596 nm, 591 nm, 591 nm, 583 nm, and 594 nm, respectively.

### 3.3. Dyeing and Fixing Process

Cotton dyeing was operated using 1002 (Roaches International Co., West Yorkshire, UK) Padding, Drying, Heat-setting and Steam Combination Apparatus. Both the dip–pad–steam process and the “one-bath–one-step” method were used for the dyeing of cotton with the azo-anthraquinone reactive dyes. Dyeing was carried out at dye concentration of 10–50 g/L with 3–20 g/L of Na_2_CO_3_. The cotton was dipped into dye solution for 1–15 min at room temperature and passed between pad rolls once. The pressure on the mangle was adjusted to give about 80% pickup. Then the cotton was dried at 50 °C for 2 min and steamed at 100 °C for 5–60 min. Finally, the dyed cotton was washed thoroughly to clear the residual sodium carbonate until the solution was neutral. The washing process was subsequently carried out using a 2 g/L solution of OP-10 at 95 °C for 10 min, followed by water until no dye was removed off, then rinsed and dried.

### 3.4. Measurement of Dye Fixation

The dye fixation (*F*) was calculated using Equation (1) [27]. The absorbance was determined using an HP 8453 UV-vis spectrophotometer at the λ_max_ of each dye, and *A*_0_, *A*_1_, and *A*_2_ of the dye liquors were measured with the same volume.

*F* = (*A*_0_ − *A*_1_ − *A*_2_)/(*A*_0_ − *A*_1_) × 100% (1)

where *A*_0_ is the absorbance of the dye bath before dyeing, *A*_1_ is the absorbance of the dye bath after dyeing, and *A*_2_ is the absorbance of the soap bath after the soaping step.

### 3.5. Measurement of Color Yield

The color yield (K/S) values of the dyed fabric were measured using an Ultra Scan XE Color Measuring and Matching Meter (Hunter Co., Reston, VA, USA) at room temperature. The color yield—ranging from a wavelength of 400 nm to 700 nm with 10 nm interval—was measured and calculated using Equation (2) [28]. All K/S values were determined at λ_max_, and average values were obtained at five different positions for each dyed fabric.

*K*/*S* = (1 − *R*)^2^/2*R* (2)

where *K* is the light absorption coefficient of the fabric, *S* is the light scattering coefficient, and *R* is the light reflectance at the maximum wavelength of the fabric.

The colorimetric properties of dyed fabric were analyzed on an Ultra Scan XE Color Measuring and Matching Meter (Hunter Co., USA) in terms of the CIELab values (L *, a *, b *, C *, h *), with a CIE standard illuminant D65 and an observer angle of 10° [29].

### 3.6. Fastness Testing

Wash fastness was assessed using a 5 g/L standard soap solution at 40 °C for 30 min in an S-1002 two-bath dyeing and testing apparatus (Roaches International Ltd., West Yorkshire, UK) according to ISO 105-C10:2006. Dry and wet rub fastness were tested according to ISO 105-X12:2001 using a Y(B)571-II crockmeter (Darong Standard Textile Apparatus Co. Ltd., Wenzhou, China). Light fastness was tested according to ISO 105-B02:1999 using YG(B)611-V light fastness tester (Darong Standard Textile Apparatus Co. Ltd., Wenzhou, China). Fading fastness was measured according to ISO 105-A05:1996 using a DigiEye color fastness tester (VeriVide Co., Leicester, UK).

## 4. Conclusions

The dyeing method and the properties of azo-anthraquinone reactive dyes on cotton have been reported. The optimum dyeing conditions in the dip–pad–steam dyeing process are as follows: dye concentration of 25 g/L, sodium carbonate of 12 g/L, dipping time of 3 min, and steaming time of 30 min with “one-dip–one-nip” process. Under these conditions, the fixation of the dyes on cotton reached up to 93.4%, the wash and rub fastness all reached grade 4 above, and the light fastness reached grade 4–5 above. Such colored cotton showed very close colorimetric properties. All these results brought very promising future applications for this kind of dyes.

## Figures and Tables

**Table 1:** Fixation and fastness properties of azo-anthraquinone reactive dyes on cotton.

Dye	F (%)	K/S	L *	a *	b *	C *	h *	Wash Fastness		Rub Fastness		Light Fastness
								Change	Stain	Dry	Wet	
DB_1_	93.4	14.0	28.3	−1.1	−20.5	20.5	266.9	4−5	4−5	4−5	4	5
DB_2_	88.4	11.2	26.8	−0.6	−19.6	19.6	268.3	4	4−5	4	4	5
DB_3_	91.8	12.8	26.4	−0.8	−19.3	19.3	267.6	4−5	4−5	4−5	4	5
DB_4_	88.2	11.0	27.0	−1.0	−18.7	18.7	266.8	4	4−5	4	4	5
DB_5_	89.9	10.2	26.9	−1.4	−15.1	15.1	264.6	4−5	4−5	4	4	4−5
